# *Cordyceps cicadae* Ameliorates Renal Hypertensive Injury and Fibrosis Through the Regulation of SIRT1-Mediated Autophagy

**DOI:** 10.3389/fphar.2021.801094

**Published:** 2022-02-10

**Authors:** Yuzi Cai, Zhendong Feng, Qi Jia, Jing Guo, Pingna Zhang, Qihan Zhao, Yao Xian Wang, Yu Ning Liu, Wei Jing Liu

**Affiliations:** ^1^ Beijing University of Chinese Medicine, Beijing, China; ^2^ Key Laboratory of Chinese Internal Medicine of Ministry of Education, Beijing Dongzhimen Hospital Addiliated to Beijing University of Chinese Medicine, Beijing, China; ^3^ Department of Nephropathy, Beijing Traditional Chinese Medicine Hospital Pinggu Hospital, Beijing, China; ^4^ Department of Nephropathy, Dongfang Hospital, Beijing University of Chinese Medicine, Beijing, China; ^5^ Department of Endocrinology Nephropathy of Dongzhimen Hospital, Beijing University of Chinese Medicine, Beijing, China

**Keywords:** hypertensive renal injury, *Cordyceps cicadae*, autophagy, SIRT1, fibrosis

## Abstract

Hypertensive renal injury is a complication of hypertension. *Cordyceps cicadae* (*C. cicadae*) is a traditional Chinese medicine used to treat chronic kidney diseases especially renal fibrosis. Autophagy is described as a cell self-renewal process that requires lysosomal degradation and is utilized for the maintenance of cellular energy homeostasis. The present study explores the mechanism underlying *C. cicadae*’s renoprotection on hypertensive nephropathy (HN). First, HN rat models were established on spontaneously hypertensive rats (SHRs). The expression of fibrosis-related protein and autophagy-associated protein was detected *in vivo*. NRK-52E cells exposed to AngII were chosen to observe the potential health benefits of *C. cicadae* on renal damage. The level of extracellular matrix accumulation was detected using capillary electrophoresis immunoquantification and immunohistochemistry. After treatment with lysosomal inhibitors (chloroquine) or an autophagy activator (rapamycin), the expression of Beclin-1, LC3II, and SQSTM1/p62 was further investigated. The study also investigated the change in sirtuin1 (SIRT1), fork head box O3a (FOXO3a), and peroxidation (superoxide dismutase (SOD) and malondialdehyde (MDA)) expression when intervened by resveratrol. The changes in SIRT1 and FOXO3a were measured in patients and the SHRs. Here, we observed that *C. cicadae* significantly decreased damage to renal tubular epithelial cells and TGFβ1, α-smooth muscle actin (α-SMA), collagen I (Col-1), and fibronectin expression. Meanwhile, autophagy defects were observed both *in vivo* and *in vitro*. *C. cicadae* intervention significantly downregulated Beclin-1 and LC3II and decreased SQSTM1/p62, showing an inhibition of autophagic vesicles and the alleviation of autophagy stress. These functions were suppressed by rapamycin, and the results were just as effective as the resveratrol treatment. HN patients and the SHRs exhibited decreased levels of SIRT1 and FOXO3a. We also observed a positive correlation between SIRT1/FOXO3a and antifibrotic effects. Similar to the resveratrol group, the expression of SIRT1/FOXO3a and oxidative stress were elevated by *C. cicadae in vivo*. Taken together, our findings show that *C. cicadae* ameliorates tubulointerstitial fibrosis and delays HN progression. Renoprotection was likely attributable to the regulation of autophagic stress mediated by the SIRT1 pathway and achieved by regulating FOXO3a and oxidative stress.

## Introduction

Hypertensive renal injury is the primary underlying disease of renal failure (Aibara et al., 2020). Tubulointerstitial fibrosis is considered to be the primary pathogenesis for progressive hypertensive nephropathy (HN) (Liu et al., 2015; Bao et al., 2018; Tao et al., 2019). Renal function and arterial pressure are regulated by the renin–angiotensin–aldosterone system (RAAS) (Francois et al., 2004). Angiotensin II (AngII), the primary effector of RAAS, can accelerate renal interstitial fibrosis progression by affecting renal hemodynamics, regulating the growth of renal tubular epithelial cells (TEC), promoting the production of inflammatory and cellular cytokines, and promoting the accumulation and degradation of the extracellular matrix (ECM) containing TGFβ1, α-SMA, FN, and Col-1 (Lu et al., 2019; Yoon et al., 2020; Zhang J.-h. et al., 2021). Recent treatment schemes for HN have primarily focused on regulating blood pressure (BP) and protecting kidney function. RAAS inhibitors, such as angiotensin-converting enzyme (ACE) inhibitors and angiotensin receptor blockers (ARBs), are considered as first-line treatments for hypertensive individuals with kidney disease. To some extent, the existing treatment methods can delay the progression of hypertension but weakly participate in controlling hypertensive renal damage (Zhang et al., 2020). The potential health effects of traditional Chinese medicine (TCM) should be explored and developed.

Autophagy (macrophage) is described as a cell self-renewal process that relies on lysosomal degradation. Physiologically, active autophagy recognizes and degrades damaged proteins, invading pathogens, and aging organelles under dystrophic and stress stimuli and subsequently releases products that are degraded for reuse to maintain cell homeostasis (Mizushima and Komatsu, 2011; Rechter et al., 2016). Autophagy consists of four phases, namely, induction, nucleation, elongation, and diffusion, and these phases are tightly regulated by different signaling pathways, such as 5′ adenosine monophosphate-activated protein kinase (AMPK), lysosomal enzymes (Chen et al., 2016), and autophagy proteins (Atgs, including LC3II). SQSTM1/p62 is an indicator of autophagic flux and acts as an autophagy receptor involved in the targeting of cargo into autophagosomes. After the fusion of autophagosomes with lysosomes, the autophagosome content, including p62, is degraded (Lim et al., 2015). Pathologically, prolonged pathogenic factors enhance autophagy induction and disrupt lysosome function, exceeding the degradative capacity in cells and contributing toward autophagic stress and possibly stagnation of autophagy (Zheng et al., 2020). ECM remodeling is the hallmark of HN. Uncontrolled ECM accumulation due to an imbalance between formation and degeneration has been implicated in renovascular fibrosis (Chen et al., 2017). The central role of autophagy in altering ECM degradation has been investigated in previous studies (Cinque et al., 2015; Eckhart et al., 2019). The contribution of autophagy to HN remains unclear; however, defects in autophagy have been associated with intracellular aging and protein and organelle damage (Guo et al., 2020), as well as disorganization of ECM components and the occurrence of tubulointerstitial fibrosis (Li, et al., 2020). Autophagy is thus considered a potential therapeutic target for HN treatment.

Redox homeostasis disorder under pathological conditions results in excessive production of reactive oxygen species (ROS) (Ornatowski et al., 2020). Oxidative stress produced by pathologic and pharmacological factors plays an important role in controlling hypertension-related diseases under physiological conditions (Guzik and Touyz, 2017). Peroxidation dysfunction and inflammation, which are identified as significant functions underlying kidney diseases, modulate the autophagy inhibition or activation and lead to cellular recycling dysfunction (Lin et al., 2019; Li et al., 2021). Accumulating evidence demonstrates that autophagy is essential to support redox homeostasis. ROS activates autophagy, which maintains cellular adaptation and reduces oxidative damage by degrading and recycling damaged macromolecules and dysfunctional organelles in cells (Ornatowski et al., 2020). On the other hand, peroxidation levels, such those as of H_2_O_2_ and AngII, in abnormal conditions may induce apoptosis via silent information regulator 2 homolog 1 (SIRT1) and other signaling pathways (Lakhani et al., 2019; Park et al., 2020; Wu et al., 2020; Huang et al., 2021). Malondialdehyde (MDA) is a major byproduct in oxidative stress that affects kidney fibrogenesis related to hypertension (Chen et al., 2019). Superoxide dismutase (SOD) is an important antioxidant enzyme that plays an indispensable role in free-radical scavenging and blocking the progression of HN (da Silva Cristino Cordeiro et al., 2018). Both have been recognized as the primary factor related to the pathogenesis of chronic kidney disease (CKD) and a cause of renal fibrosis.

SIRT1 is ubiquitously expressed in the human body, including in kidney cells such as podocytes, glomerular mesangial cells, and tubular cells. Using deacetylating substrates, SIRT1 plays a role in regulating autophagy, oxidative stress, energetic homeostasis, and apoptosis (Zhang et al., 2017; Liu et al., 2018; Xu et al., 2020). Renal SIRT1 is cytoprotective and is correlated to BP regulation and sodium balance (Hao and Haase, 2010). Recent studies have focused on the role of SIRT1 in HN development and progression, primarily by protecting tubular cells from cellular stresses (Guclu et al., 2016; Huang X. et al., 2020; Martinez-Arroyo et al., 2020).

FOXO3a, a major downstream molecule of SIRT1, belongs to the fork head box O (FOXO) family of transcription factors. It has been suggested that the FOXO family serves as a SIRT1 deacetylate member that affects downstream pathways that control autophagy (Daitoku et al., 2011; Tang, 2016). However, SIRT1 largely influences FOXO3a-mediated transcription during oxidative stress and cell survival by controlling FOXO3a deacetylation (Kobayashi et al., 2005). Resveratrol (RES), a well-known SIRT1 activator and oxidative stress inhibitor, ameliorates renal tubular damage in DN by upregulating FOXO3a transcriptional activity and reinforces resistance to oxidative damage (Jiang et al., 2020). RES and its active component also regulate autophagy via the SIRT1/FOXO3a pathway in many diseases (Shi et al., 2012; Ni et al., 2013). Emerging evidence indicated that *C. cicadae* elevated the SIRT1 expression against kidney injury or renal interstitial fibrosis (Huang YS. et al., 2020). Additionally, RES treatment ameliorates renal function and glomerulosclerosis and increases SIRT1 deacetylase activity, subsequently decreasing the expression of acetylated FOXO3a and inhibiting the oxidative stress caused by hyperglycemia both *in vivo* and *in vitro* (Wang et al., 2017). Thus, this study aims to determine whether *C. cicadae* imparts a protective effect against NH via the SIRT1/FOXO3a/ROS pathway.

*C. cicadae* is a TCM that belongs to the family Cordycipitaceae, which is parasitic on *Cicada flammata* larvae. It has been utilized in the treatment of various diseases and to relieve exhaustion. Pharmacological studies have shown that the fungus contains biologically active chemical substances including nucleosides, cordycepic acid, cordycepin, beauvericin, and myriocin (Sun et al., 2017). *C. cicadae* has been historically utilized for liver and kidney protection, analgesia–antipyresis, blood fat reduction, and its antitumor activities (Qin et al., 2020). It is well known that the active ingredients extracted from *C. cicadae* are effective in ameliorating CKD induced by diabetes or hypertension. (Zheng et al., 2018; Huang X. et al., 2020; Liu et al., 2014). Evidence also suggests that *C. cicadae* relieved acute kidney injury through the inhibition of oxidative stress and inflammation (Deng et al., 2020). Based on our previous studies, we estimated that *C. cicadae* might protect renal functions against kidney fibrosis by alleviating renal autophagic stress through the regulation of the SIRT1/FOXO3a/ROS pathway. This hypothesis was assessed in spontaneously hypertensive rats (SHRs) and in AngII-cultured primary TECs.

## Materials and Methods

### Human Renal Biopsy Samples

All clinical data derived from 25 patients (age range: 50–60 years) of the Affiliated Hospital of Guangdong Medical College (Zhanjiang, Guangdong, China) were de-identified. We searched for stored former kidney biopsy samples collected from December 2015 to December 2020. These kidney tissue specimens were obtained from patients diagnosed with biopsy-proven hypertensive renal injury (*n* = 11). The inclusion criteria of the control group were patients with mild urinary protein excretion or hematuria only and biopsy-proven minimal change (*n* = 14).

### Animals

Thirty male SHRs (age: 8 weeks old; weight range: 170–210 g) and eight Wistar-Kyoto (WKY) rats (age: 8 weeks old; weight range: 170–210 g) were obtained from Beijing Vital River Co., Ltd. (Beijing, China) and housed under controlled laboratory conditions (25 ± 2°C temperature, 60 ± 1% humidity, and 07:00–19:00 light and 19:00–07:00 dark cycle) with *ad libitum* access to water. The experiments were conducted according to the *Guide for the Care and Use of Laboratory Animals* (eighth edition) (National Academies of Sciences, Engineering, and Medicine) and approved by the ethics committee of Beijing University of Chinese Medicine. Four groups were used: (1) the control WKY rats (*n* = 8), (2) SHRs (*n* = 10), (3) SHRs that received intraperitoneal injection of RES once a day (40 mg/kg/day, *n* = 10), and (4) SHRs treated with *C. cicadae* once a day (4 g/kg/day, *n* = 10). After overnight fasting on the 28th week, the animals were sacrificed, and renal tissue specimens were isolated for further analysis.

### Detection of Urinary and Plasmatic Parameters

Blood plasma was collected via the abdominal aorta and then centrifuged at 3,500 rpm for 15 min (4°C). The blood samples were stored at −80°C until use. Twenty-four-hour urine samples were collected from the rats that were situated in a metabolic cage on the 14th and 28th weeks. Urinary creatinine levels were measured using an enzyme-linked immunosorbent assay (ELISA) kit (C011-2-1, Nanjing Jiancheng Bioengineering Institute, Nanjing, China); urinary albumin levels were measured using an ELISA kit (ab108789, Abcam, Cambridge, MA, United States); and levels of urinary β2-MG were measured using an ELISA kit (RKM100, R&D Systems, Minneapolis, MN, United States). The urinary kidney injury molecule-1 (KIM-1) concentration was measured using an ELISA kit (ab119597). All protocols were performed following the manufacturers’ instructions.

### Renal Histological Examination

The paraffin-embedded kidneys were sliced into 3-μm-thick sections and dewaxed in a xylene reagent tank, and this was followed by rehydration across an ethanol gradient. Hematoxylin and eosin (H&E) staining was conducted to assess the histological changes. Masson’s trichrome staining (Masson) was used to evaluate fibrosis. The prepared sections were stained using a kit for Masson’s trichrome staining (Nanjing Jiancheng Biological Reagent Co., Ltd., Nanjing, China). Images were captured using an Olympus BX60 microscope (Olympus, Tokyo, Japan) and a Zeiss optical microscope (Germany) equipped with a ZEN 2.3 (blue edition) image-capture software (Carl Zeiss Microscopy GmbH, Jena, Germany). Image-Pro Plus (IPP) 6.0 software (Media Cybernetics, United States) was utilized to estimate the degree of interstitial fibrosis. The above methods are described in detail in our previous study (Huang YS. et al., 2020).

### Preparation of the Reagents and the *C. cicadae*-Containing Serum

*C. cicadae* was obtained from the Zhejiang BioAsia Pharmaceutical Co., Ltd. (Pinghu, Zhejiang, China). RES, chloroquine (CQ), and rapamycin (RAP) were obtained from Sigma-Aldrich (St. Louis, MO, United States).

Sprague-Dawley rats weighing 250–300 g were randomly divided into negative control and treatment groups. The treatment group animals were treated with *C. cicadae* or distilled water (2 ml/day) as previously described. After a 1-week treatment, blood samples were collected from the abdominal aorta and centrifuged. Serum samples from all individual animals of each group were pooled and filtered through a 0.22 μm filter membrane. Medicated serum containing *C. cicadae* (CMS) was employed in the cell experiment.

### Cell Culture and Treatment

NRK-52E cell lines were provided by the Institute of Nephrology of the General Hospital of the Chinese People’s Liberation Army (Beijing, China). The cells were cultured at Dongzhimen Hospital of Beijing University of Chinese Medicine (Beijing, China) and supplemented with 10% fetal bovine serum (FBS, Gibco, United States) in a humidified 5% CO_2_ atmosphere at a temperature of 37°C. The cells were subcultured every 2 to 3 days. In several experiments, the NRK-52E cells were incubated with AngII (10^–7^ mM), CMS (10%), and RES (25 μM). All cells were harvested for further analysis.

### Cell Viability Assay

The viability of the NRK-52E cells treated with AngII (Sigma-Aldrich) was assessed using Cell Counting Kit-8 (CCK-8, Dojindo Laboratories, Kumamoto, Japan). The cells at a density of 5 × 10^3^ cells per well were seeded into 96-well plates and then treated with AngII (10^–4^, 10^–5^, 10^–6^, 10^–7^, 10^–8^, and 10^−9^ M) for various duration (24 and 72 h). Ten microliters of CCK-8 solution was added into each well and then cultured for another 1–4 h. Finally, the optical density (OD) value at a wavelength of 450 nm was measured to determine cell viability (Thermo Scientific, United States). The assay was repeated thrice. A CCK-8 assay was used to determine the safe and effective AngII concentration for the experiment. Different concentrations of CMS (10, 15, 20%) and RES (10, 25, 50, 100, 150 μM) were detected as above.

### Detection of Cell Injury

There were four groups in the experiment: a control group, an AngII group, a CMS group, and a RES group. The cells were cultured as earlier described, and KIM-1 and neutrophil gelatinase-associated lipocalin (NGAL) abundance in the supernatant was determined by ELISA kits obtained from R&D Systems (DLCN20, Minneapolis, MN, United States) and Abcam (ab119597). All of the operational procedures were performed in accordance with the manufacturers’ instruction.

### MDA and SOD Detection

After treatment, the renal tissues or supernatant were collected, and MDA and SOD were quantified using an MDA assay kit (S0131, Beyotime Institute of Biotechnology, Jiangsu, China) and a SOD assay kit (S0101, Beyotime Institute of Biotechnology, Jiangsu, China) following the manufacturer’s instruction.

### Western Blot and Densitometric Analysis

Total protein from cells of rat kidney tissues was extracted by centrifugation at 12,000 rpm at 4°C for 20 min. The resulting supernatant was collected and used to determine total protein concentration using the BCA assay. Supernatants of various samples were heated for 10 min at 95°C in a sample loading buffer and later separated by SDS-PAGE and then transferred to nitrocellulose membranes. The membranes were blocked for 1 h with 5% nonfat milk in TBST buffer. To perform immunodetection, the blots were incubated at 4°C overnight with the following primary antibodies: anti-LC3II (ab51520), anti-SQSTM1/p62 (ab109012), beclin-1 (ab62557), anti-α-SMA (ab32575), anti-TGF-β1 (ab92486), anti-FN (ab2413), anti-Col-1 (ab34710), and anti-GAPDH antibody (10494-1-AP, ProteinTech, Rosemont, PA, United States). Except for the anti-GAPDH antibody, all primary antibodies were obtained from Abcam and employed at a 1:1,000 dilution. The peroxidase-linked secondary antibody (SA00001-2, ProteinTech, Rosemont, PA, United States) was diluted at 1:5,000. Then, the membranes were visualized with an ECL advanced kit, and quantitation of protein bands was performed using the ImageJ software (NIH, Bethesda, MD, United States). The result of absorbance measurements and the grey values obtained from the densitometric analysis were expressed as means ± standard deviations (SDs) of the three determinations for each sample.

### Capillary Electrophoresis Immunoquantification

Whole-cell protein was obtained for quantitative capillary isoelectric immunoassay. Protein levels were assessed using a capillary-based automated electrophoresis immunoquantification instrument (ProteinSimple, San Jose, CA, United States) following the manufacturer’s standard instruction. Here, 3 μl of protein extract (final concentration: 1 μg/μl) was loaded with the following antibodies: anti-SIRT1 antibody (sc15404, Santa Cruz Biotechnology, Santa Cruz, CA, United States), anti-GAPDH antibody (10494-1-AP, ProteinTech), and anti-FOXO3a antibody (12829, Cell Signal Technology, Inc., United States). The run conditions were as recommended previously. Compass software (ProteinSimple ver. 3.1.8) was utilized to calculate and measure the immunoblots.

### Immunostaining Analysis

Immunohistochemical and immunofluorescence analyses of samples were performed as described. Rabbit anti-SIRT1 (sc15404, Santa Cruz Biotechnology, Santa Cruz, CA, United States), rabbit anti-FOXO3a antibody (12829, Cell Signaling Technology, CA, United States), rabbit anti-α-SMA antibody (ab32575, Abcam, Cambridge, MA, United States), rabbit anti-LC3II antibody (ab51520, Abcam, Cambridge, MA, United States), rabbit anti-SQSTM1/p62 (ab109012), and fluorescein isothiocyanate-labeled goat anti-rabbit IgG (sc-2012, Santa Cruz Biotechnology, Santa Cruz, CA, United States) were used in the immunostaining assay for kidney tissues. Rabbit anti-FOXO3a antibody (12829, Cell Signaling Technology, CA, United States), rabbit anti-SIRT1 (2977886, Millipore, Billerica, MA, United States), rabbit anti-LC3II antibody (ab51520, Abcam, Cambridge, MA, United States), rabbit anti-SQSTM1/p62 (ab109012), rabbit anti-α-SMA antibody (ab32575, Abcam, Cambridge, MA, United States), and Alexa Fluor 488 donkey anti-rabbit IgG (Invitrogen, Carlsbad, CA, United States) were used for immunostaining the cells. The nuclei of the cells were stained with DAPI. Immunopositive signals were detected using a confocal microscope (Leica Microsystems, Wetzlar, Germany) or an Olympus BX60 microscope (Olympus, Tokyo, Japan). The acquired images were analyzed using the IPP 6.0 software.

### Statistical Analysis

Statistical analysis was conducted using the GraphPad Prism ver. 9.00 statistical software (SAS Institute, Abacus Concept, Inc., Berkeley, CA, United States). Results are shown as means ± SDs. Differences among groups were examined using one-way ANOVA, which was then followed by Bonferroni multiple-comparison test. Comparison among treatment groups was performed at a significance level of *p* < 0.05.

## Results

### *C. cicadae* Alleviated Renal Injury and Fibrosis of the SHRs

**FIGURE 1:**
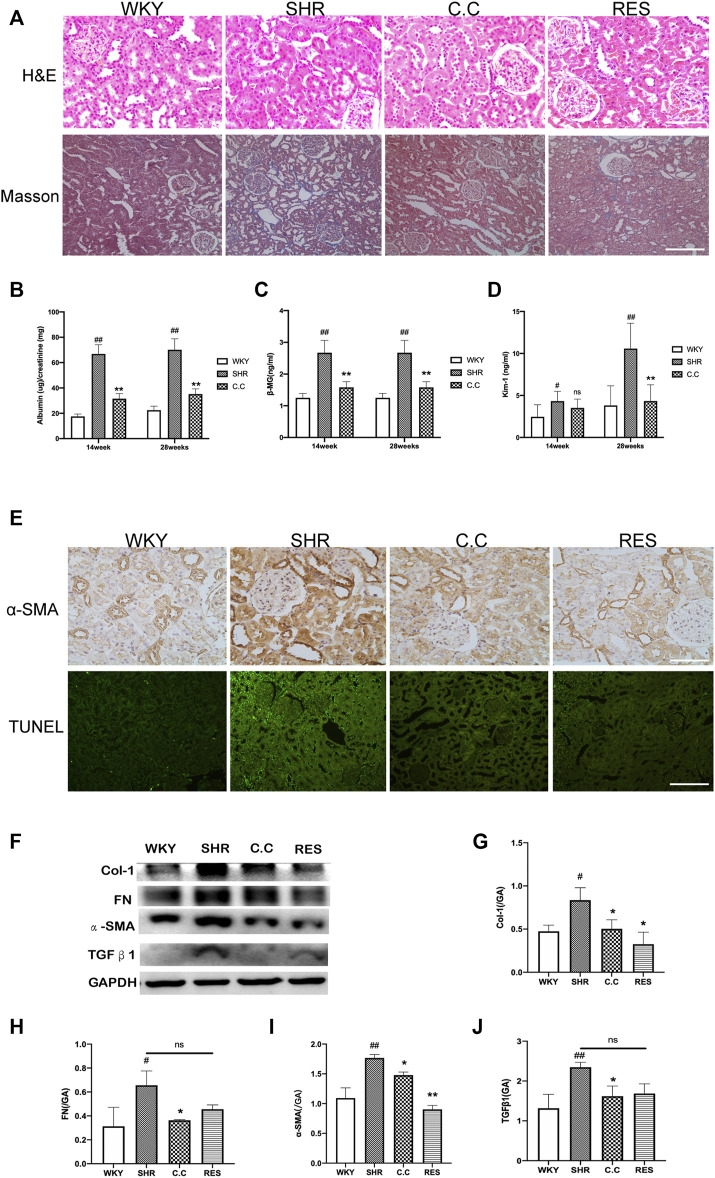
*C. cicadae* alleviated SHR renal injury and fibrosis. **(A)** H&E staining (bar = 100 μm) and Masson’s trichrome staining (bar = 40 μm). **(B)** *C. cicadae* treatment significantly decreases ACR levels after 14 and 28 weeks. **(C)** *C. cicadae* treatment significantly downregulates β2-MG expression after 14 and 28 weeks. **(D)** *C. cicadae* treatment significantly reduced β2-MG level at the 28th week. **(E)** The representative images and statistical graph of IHC staining for α-SMA (×200). Bar = 100 μm. Apoptosis in rat renal tubules of various groups was evaluated by TUNEL assay. Scale bar: 40 μm. **(F)** Effect of *C. cicadae* and RES on TGFβ1, α-SMA, FN, and Col-1 expression. **(G–J)** The relative intensities of fibrosis-related protein in kidneys were calculated after normalization against GAPDH. Data were presented as mean ± SD, *n* = 10 rats per group. For the WKY group vs the SHR group, #*p* < 0.05, ##*p* < 0.01. For the SHR group vs the *C. cicadae* group, *p < 0.05, **p < 0.01. N.S. No significance.

We determined the impact of *C. cicadae* on renal damage and fibrosis after 28 weeks of induction *in vivo*. No significant histopathological differences among the four H&E-stained groups were detected. Masson’s trichrome staining revealed that the SHR group developed fibrotic changes and severe renal interstitial fibrosis; in addition, *C. cicadae* significantly decreased collagen deposition (Figure 1A). A comparison of the WKY and SHR groups indicated significant differences in the albumin/creatinine ratio (ACR), as well as KIM-1 and β2-MG levels. Treatment with *C. cicadae* significantly downregulated ACR, KIM-1, and β2-MG levels by the 28th week, whereas no marked changes in KIM-1 level were detected on week 14 (Figures 1B–D). We also assessed the number of apoptotic kidney cells using the terminal transferase-mediated biotin dUTP nick-end labeling (TUNEL) assay, and immunohistochemical staining revealed that with prolonged AngII treatment, the SHR group showed a gradual increase in collagen fiber (α-SMA) expression and rate of renal intrinsic cell apoptosis, whereas ECM deposition and apoptotic TEC count significantly decreased with *C. cicadae* treatment after 28 weeks (Figure 1E). Similarly, the ECM accumulation was evaluated using western blot, and *C. cicadae* downregulated the TGFβ1, α-SMA, FN, and Col-1 expression and protected the hypertension-injured kidney tissue from progressive fibrosis (Figures 1F–J).

### *C. cicadae* Attenuated AngII-Treated NRK-52E Cell Injury and Fibrosis

**FIGURE 2:**
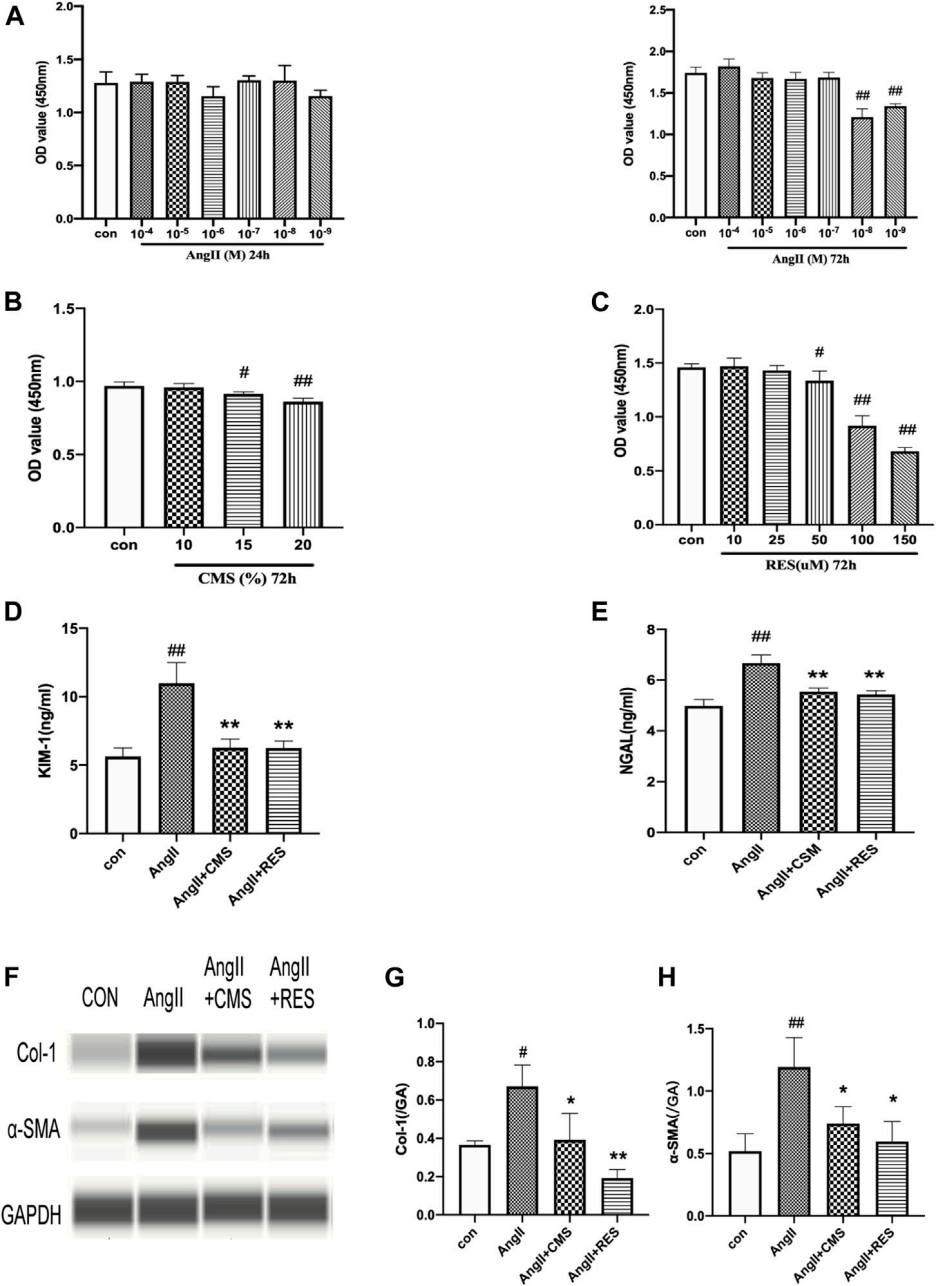
CMS attenuated AngII-associated injury and inhibited ECM accumulation in rat renal tubular epithelial cells. **(A)** NRK-52E cells were exposed to various concentrations of AngII for 24 and 72 h. **(B)** Effects of CMS on viability for 72 h. **(C)** Effects of RES on NRK-52E cell viability for 72 h. **(D**,**E)** Effect of CMS on NRK-52E cell injury. The level of cell injury was examined by KIM-1 and NGAL ELISA kits. **(F)** Effect of CMS on the expression of Col-1 and α-SMA. **(G**,**H)** The relative intensities of fibrosis-related protein in cells were calculated after normalization against GAPDH. Data are expressed as the mean ± SD of three separate experiments. ^#^ *p* < 0.05, ^##^ *p* < 0.01 compared with control cells. *p < 0.05, **p < 0.01, compared with the model group.

AngII was employed to simulate hypertensive renal injury. This study selected NRK-52E rat renal epithelial cells as the model of renal damage of hypertension. A dose-related change in cell activity and viability was observed in the NRK-52E cells exposed to various concentrations for 24 and 72 h. As shown in Figure 2A, the cell viability significantly decreased at an AngII concentration of 10^–8^ M (*p* < 0.01), and 10^–7^ M of AngII was chosen to be the optimal model concentration. We also detected the effect and safe concentration of *C. cicadae* by diluting the medicated serum. Figure 2B indicates that the NRK-52E cells remained in a steady growth environment at 10% *C. cicadae* serum. Eventually, we selected the 10% concentration in the following study. By the same method, the concentration of RES was confirmed to be 25 μM (Figure 2C).

Renal tubular epithelial cell damage was evaluated based on supernatant KIM-1 and NGAL levels, which were significantly upregulated in the AngII group relative to the control group (Figures 2D,E). However, *C. cicadae* treatment significantly downregulated KIM-1 and NGAL expression by 46% and 15%, respectively, relative to the model group. These findings coincided with the changes in kidney tissues obtained in the SHRs. The ECM accumulation was assessed using capillary electrophoresis immunoquantification (Figure 2F). α-SMA and Col-1 were significantly upregulated in the AngII group relative to the control group, which were reduced with *C. cicadae* treatment (Figures 2G,H).

### *C. cicadae* Ameliorated Hypertensive Renal Fibrosis by Inhibiting Autophagic Stress

**FIGURE 3:**
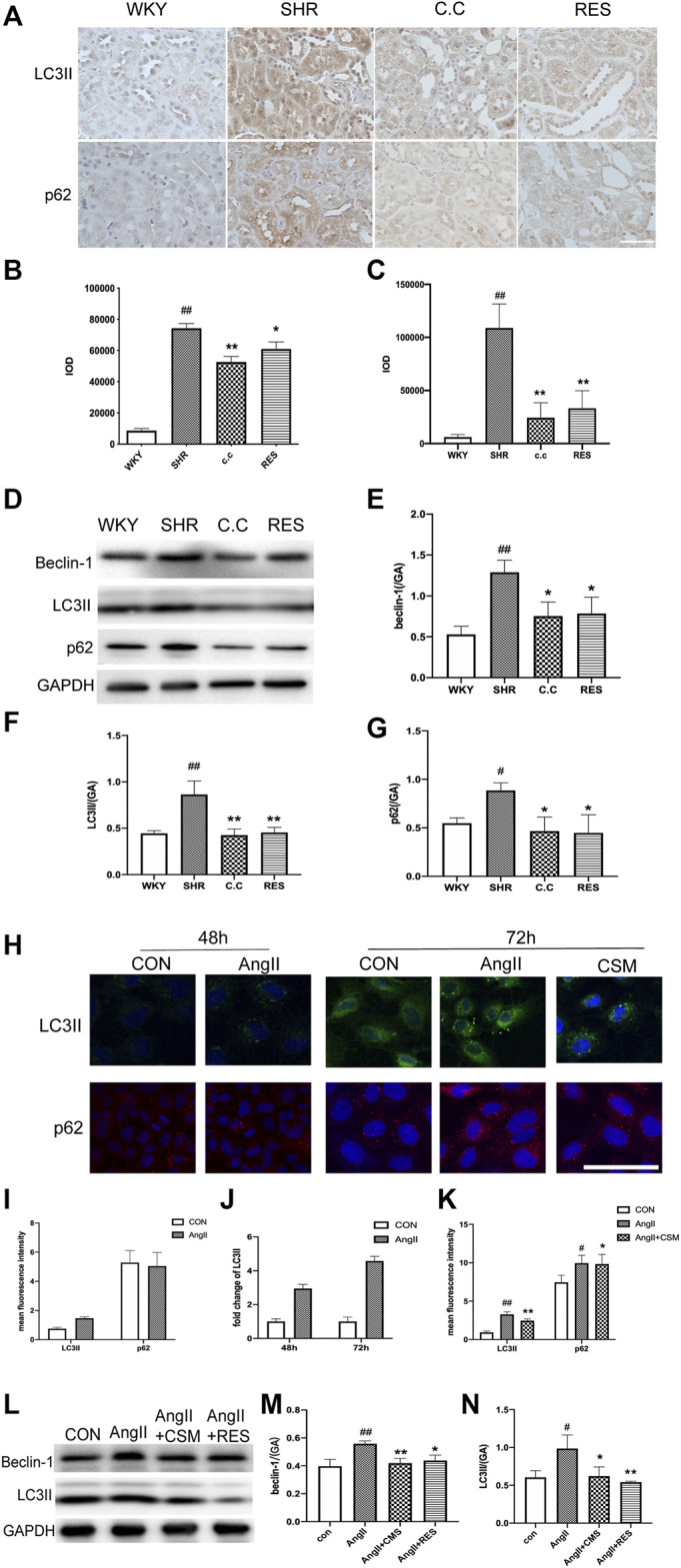
*C. cicadae* and RES regulated autophagic stress in hypertensive kidney damage. **(A)** IHC staining result (×400) of LC3II and p62 in paraffin sections. Bar = 50 μm. **(B**,**C)** Statistical results of IHC staining of LC3II and p62, respectively. The value of mean optical density (IOD) obtained by IPP software. **(D)** Expression of beclin-1, LC3II, and p62. The results of evaluation are shown in **(E–G)**. ^#^ *p* < 0.05, ^##^ *p* < 0.01: compared with controls. *p < 0.05, **p < 0.01: relative to the model group. N.S. No significance. **(H)** Immunofluorescence images show the levels of LC3II (green channel) and the levels of p62 (red channel) for 48 and 72 h. Bar = 20 μm. **(I–K)** Statistical results of immunofluorescence staining of LC3II and p62. The value of IOD obtained by IPP software. **(L)** Expression of beclin-1 and LC3II. The results of evaluation are shown in **(M)** and **(N)**. ^#^ *p* < 0.05, ^##^ *p* < 0.01: compared with controls, respectively. *p < 0.05, **p < 0.01: relative to the model group, respectively.

Autophagic stress means an increase in the autophagic flow. This process may be due to the generation of too many autophagic vesicles that cannot be degraded or a change in the autophagic flux due to problems with autophagic degradation. We next investigated whether *C. cicadae* could reduce fibrosis protein expression and suppress ECM accumulation by regulating autophagy. The autophagic markers LC3II and p62 and autophagosomal formation were employed to evaluate the condition of autophagy in the SHR kidney tissue using immunohistochemical staining. LC3II and p62 protein expression was upregulated in hypertensive renal damage, which was indicative of autophagic activation (Figures 3A–C). Similar observations were obtained using western blot (Figure 3D), whereas *C. cicadae* downregulated LC3II, beclin-1, and p62 expression (Figures 3E–H). In addition, we observed increased LC3II expression along with blocked p62 degradation for the 72-h-treated AngII NRK-52E cells, indicating enhanced autophagosome synthesis and the defect of LC3II-mediated protein degradation (Figures 3I–L). As shown in Figures 3M,N, the expression of LC3II and beclin-1 was suppressed after the *C. cicadae* treatment, indicating that *C. cicadae* inhibited autophagy.

**FIGURE 4:**
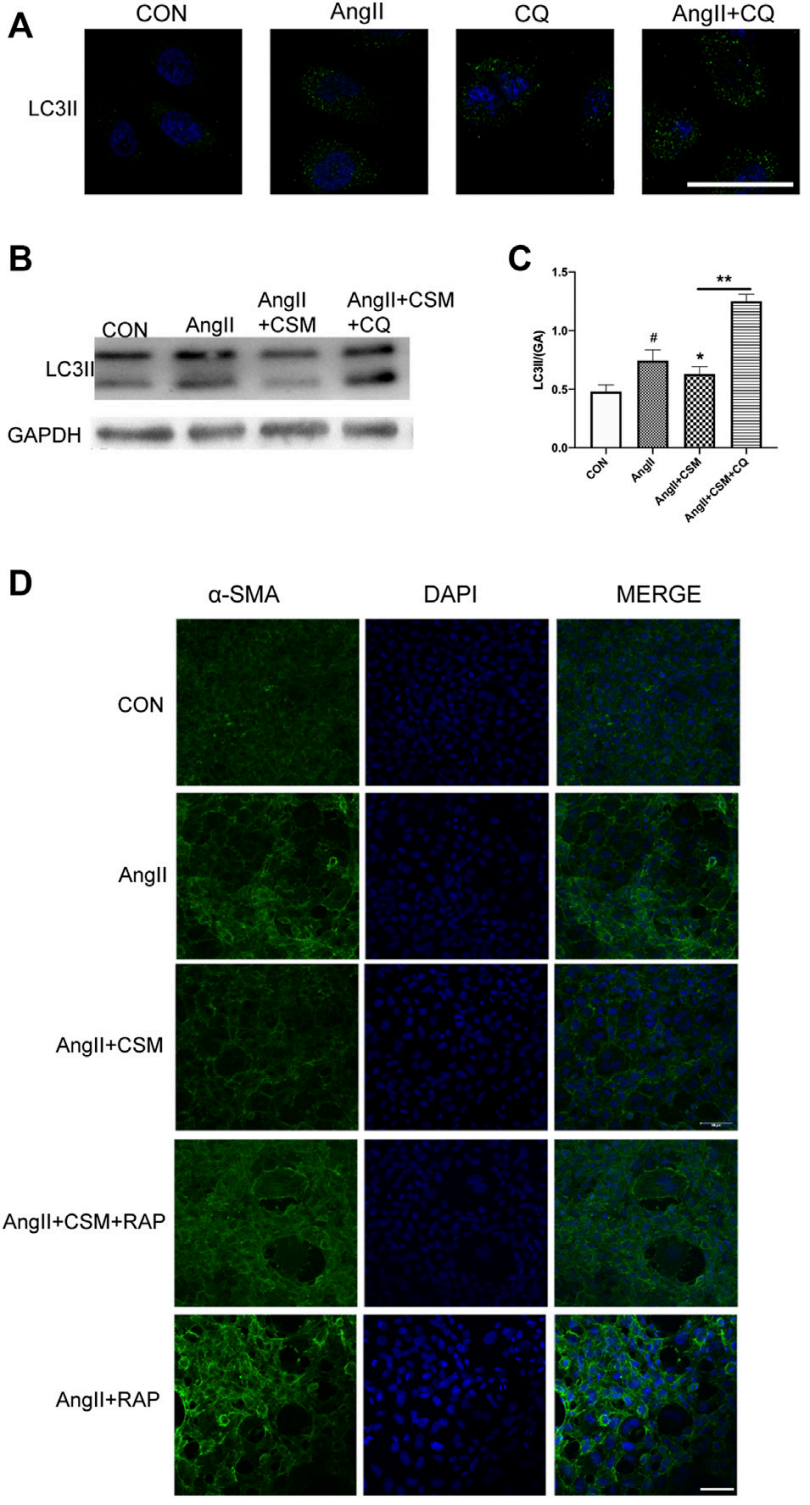
CMS attenuated fibrosis via regulating autophagic stress *in vitro*. **(A)** NRK-52E cells were treated with AngII for 72 h, in the presence or absence of CQ (10 μM). **(B)** CMS regulated autophagic stress. LC3II protein expression was assessed by western blotting. **(C)** Statistical results of LC3II. **(D)** Immunofluorescence images show the levels of α-SMA (green channel) for 72 h. NRK-52E cells were exposed to AngII, with or without CMS and in the presence or absence of RAP (50 nM) for 72 h. The fluorescence intensity was detected with a laser confocal microscope, bar = 20 μm. ^#^ *p* < 0.05: relative to untreated cells. *p < 0.05, **p < 0.01: relative to the model group.

To further investigate the impacts of autophagy on the renal fibrosis process, we treated the NRK-52E cells with lysosomal inhibitor CQ. As shown in Figure 4A, the CQ exacerbated autophagic activation caused by AngII. We also found that the autophagic inhibition effect of *C. cicadae* was blocked after being exposed to CQ for 72 h (Figures 4B,C). Then, the autophagy activator RAP was used. The immunofluorescence assay showed that the expression of α-SMA was reversed by the *C. cicadae* treatment, while no reversed effect was achieved due to the RAP treatment (Figure 4D). In addition, the NRK-52E cells treated with *C. cicadae* as well as RAP suppressed the antifibrotic effect of *C. cicadae*. These results indicated that *C. cicadae* ameliorated hypertension-induced renal fibrosis by suppressing autophagic induction, reducing autophagy vesicle formation, and suppressing autophagy stress.

### *C. cicadae* Increased SIRT1/FOXO3a Expression and Decreased Oxidative Stress in Hypertensive Renal Damage

**FIGURE 5:**
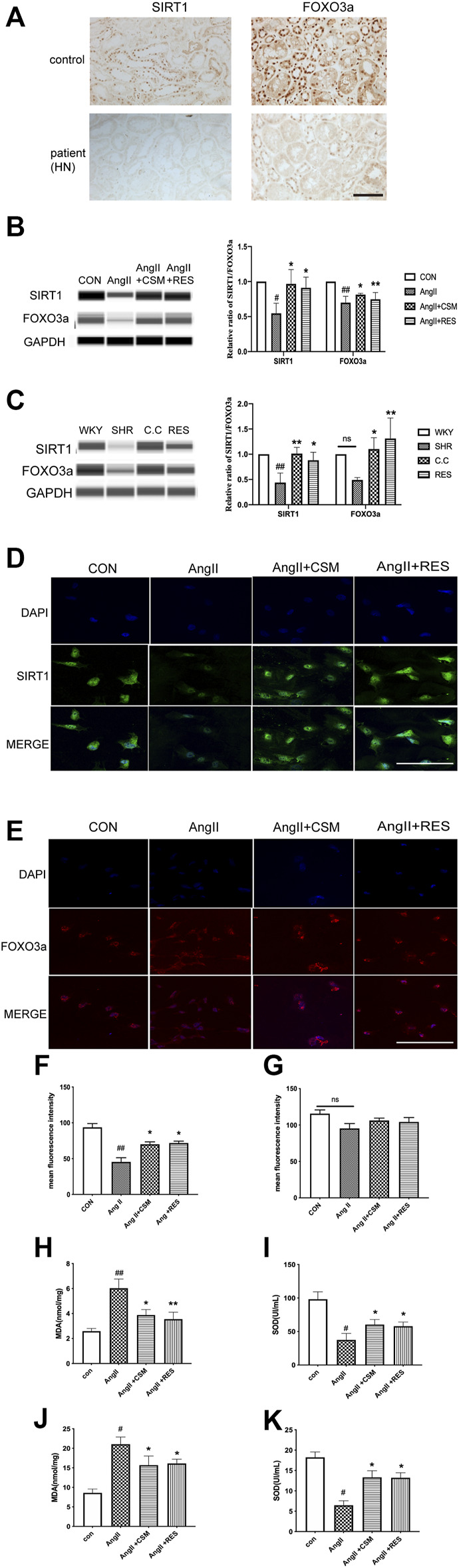
Experimental validation of the SIRT1/FOXO3a/ROS signaling pathways *in vivo* and *in vitro*. **(A)** Downregulated SIRT1 and FOXO3a existed in HN patients (bar = 40 μm). **(B)** Capillary electrophoresis immunoquantification of SIRT1 and FOXO3a on NRK-52E cells in each group. **(C)** Capillary electrophoretic analysis of SIRT1 and FOXO3a expression in the rat renal cortex of various study groups. **(D**,**E)** CMS regulated SIRT1 (green channel) and FOXO3a (red channel) signaling pathways on NRK-52E cells (bar = 20 μm). **(F**,**G)** Statistical results of immunofluorescence staining of SIRT1 and FOXO3a, respectively. Densitometry was conducted, and the ratio of SIRT1 or FOXO3a to GAPDH was expressed as fold changes relative to the control. **(H**,**I)** The content of intracellular MDA and SOD in different treatment rats. **(J**,**K)** The activity of MDA and SOD in different treatment cells. #*p* < 0.05, ##*p* < 0.01: relative to the control group. *p < 0.05, **p < 0.01: relative to the model group. N.S. No significance.

To assess clinical relevance, we determined SIRT1/FOXO3a expression levels in human renal biopsy samples obtained from normotensive health controls as well as patients with HN. The result of the immunohistochemistry showed that higher levels of SIRT1 and FOXO3a existed in healthy subjects, while significant downregulation was seen in the HN patients (Figure 5A). Furthermore, there was a positive correlation between SIRT1 and FOXO3a. Thus, SIRT1/FOXO3a might play an important role in HN.

Then, the SIRT1 and FOXO3a expression was determined using capillary electrophoresis immunoquantification in the SHRs and NRK-52E cells. We found that the SIRT1 and FOXO3a expression was low in all of the model groups, while *C. cicadae* upregulated the SIRT1 and FOXO3a levels in hypertensive renal damage (Figures 5B,C). Similar results were obtained using *in vitro* immunofluorescence, in which *C. cicadae* induced an increase in the fluorescence intensity of SIRT1 in NRK-52E cells compared with the AngII group (Figures 5D,F). However, the immunofluorescence images showed that no significant change existed in the FOXO3a expression (Figures 5E,G). This might have been related to the nuclear translocation of FOXO3a (Supplementary Figure 1A). MDA and SOD regulate the production of ROS and are related to oxidative stress intimately. The trend of the MDA in the SHRs and AngII-treated NRK-52E cells was significantly stimulated compared to the control, while it was suppressed by *C. cicadae* (Figures 5H,J). In contrast, *C. cicadae* restored the activity of SOD in hypertensive renal damage (Figures 5I,K).

### *C. cicadae* Might Contribute to Renal Fibrosis and Injury via SIRT1/FOXO3a/ROS-Mediated Autophagy

Previous research revealed that *C. cicadae* induced fibrosis and apoptosis in SHRs by inhibiting the SIRT1/p53 pathway. Based on the fact that SIRT1/FOXO3a signaling also plays a major role in autophagy regulation, we investigated whether this pathway is associated with renal fibrosis and hypertension-induced autophagic stress both *in vivo* and *in vitro*. RES, a natural activator of SIRT1 that releases oxidative stress, has been reported in many studies. Consistent with our previous study, we found that RES increased the SIRT1 expression *in vivo* and *in vitro*. In addition, RES treatment upregulated FOXO3a while SIRT1 was activated. These results indicated that AngII suppressed the expression of SIRT1 and FOXO3a on the renal tubular epithelial cells, and the *C. cicadae* treatment reversed the downregulation equal to the RES (Figures 5A–C). Similar results were obtained by *in vitro* immunofluorescence, wherein RES or *C. cicadae* application increased the fluorescence intensity of SIRT1 in NRK-52E cells compared with the AngII group (Figures 5D,F). The immunofluorescence images showed an upward tendency, but no significant change existed in the FOXO3a expression compared with the AngII group (*p* > 0.05) (Figures 5E,G). Moreover, detection of the peroxidation levels indicated that RES also downregulated the oxidative stress levels (Figures 5H–K). The result indicated that the stimulated SIRT1 and suppressed peroxidation contributed to renal fibrosis and injury. The therapeutic effect of *C. cicadae* was realized through the SIRT1 pathway.

In the current study, the expression of the renal fibrosis-related protein was also evaluated. As shown in Figure 1, compared with *C. cicadae*, the RES treatment showed a downward regulation of α-SMA, Col-1, TGFβ1, and FN. Interestingly, western blot and immunostaining analyses revealed that RES and *C. cicadae* significantly disrupted autophagic induction, which is characterized by downregulated LC3II and beclin-1 expression (Figure 3) in hypertensive renal injury. Moreover, *C. cicadae* and RES exhibited downregulated levels of p62, indicating autophagic flux restoration (Figure 3). These results indicated that *C. cicadae* inhibited fibrosis processing by regulating autophagy in the NRK-52E cells or SHRs equal to RES. In addition, this stimulated SIRT1/FOXO3a and suppressed the oxidative stress levels that contributed to autophagic stress. Therefore, *C. cicadae* might enhance the antifibrotic effects in HN and regulate autophagy by regulating the SIRT1/FOXO3a/ROS signaling pathway.

## Discussion

Based on the current findings, our research revealed the antifibrotic and anti-injury effects of *C. cicadae* and its potential mechanisms. The new findings were as follows. (1) *C. cicadae* ameliorated renal injury and kidney fibrosis both *in vitro* and *in vivo*. (2) Autophagic stress existed in hypertensive renal damage. (3) *C. cicadae* suppressed the deposition of ECM markers by the inhibition of autophagic vesicles and the alleviation of autophagy stress. (4) *C. cicadae* regulated autophagy via the SIRT1 pathways, which might be achieved by regulating FOXO3a and oxidative stress. These results highlight a potential therapeutic strategy against hypertensive renal fibrosis and injury by natural medicine.

Hypertension is considered a major risk factor in the development of hypertensive renal injury. RAAS dysregulation contributes to the pathogenesis of cardiovascular and renal disorders. The majority of renal disorders result in renal fibrosis. Previous studies have demonstrated that the proliferation of epithelial and glomerular mesangial cells is stimulated by AngII and induces ECM deposition, which consequently causes additional renal damage (Grace et al., 2012; Ge et al., 2020; Liu et al., 2021). Here, the expression of various tubular injury markers, including KIM-1 and NGAL, was significantly upregulated in the AngII group relative to the control. In addition, AngII promoted ECM deposition, which contributes to fibrosis processing. SHRs provide an opportunity to study essential hypertension, as the natural progression of hypertension and organ damage in SHRs, including in the kidneys, is remarkably similar in humans. A previous study showed that SBP was higher in the SHR group than in the WKY, which was coupled with changes in BP and kidney function indicators (24-h urine albumin and ACR) (Huang X. et al., 2020). These results are supported by the current research that showed that the expression of TGFβ1, α-SMA, FN, and Col-1 was significantly elevated in the SHR group.

Autophagy is a cellular process involving bulk degradation of cytoplasmic components; in addition, its persistent activation is largely involved in renal damage (Bao et al., 2018). Renal fibrosis pertains to the common pathway associated with end-stage renal disease. Previous studies have demonstrated that autophagy in disease pathogenesis is complicated and related to both physiological and pathological regulation (Livingston et al., 2016; Wang et al., 2020). The present study observed a higher number of autophagosomes and autophagic stress existing in cells and rats, which was coupled with ECM deposition in interstitial hypertensive renal injury. However, *C. cicadae* treatment rescued the tubular epithelial cells from fibrosis processing and suppressed autophagic stress. Lysosomal-mediated diffusion systems are a key step of autophagy degradation, and a lysosomal inhibitor was used to examine the lysosomal function *in vitro*. The result suggested that the stability of lysosome was interrupted when exposed to AngII. Further elucidation demonstrated that lysosomal degradation also changed phase in this study. Hence, the activator of autophagy was used to investigate the potential mechanism. Here, we observed that *C. cicadae* downregulates the expression of LC3II and beclin-1 autophagosomal markers, which are two key factors that fuel the autophagic process, and it downregulates the expression of p62. In addition, both previous (Huang YS. et al., 2020) and current investigations revealed the inhibitory effect of *C. cicadae* on α-SMA expression, whereas RAP suppressed renoprotective activity. We considered mediated autophagy as a key point for therapeutic *C. cicadae* administration to inhibit ECM, thus blocking renal fibrosis.

FOXO3a regulates autophagy in various biological characteristics, including inflammation, apoptosis, oxidative stress, and aging (Liu et al., 2017; Fitzwalter and Thorburn, 2018; Galati et al., 2019; Ali et al., 2020; Xu et al., 2021). This activity is regulated by posttranslational modifications, including phosphorylation, acetylation, and ubiquitination (Daitoku et al., 2011). Various combinatorial drug treatments upregulate autophagy-related gene expression in rats via FOXO3a activation (Li et al., 2020; Zhu et al., 2020). Evidence has also indicated that FOXO3a participates in the regulation of lysosomal function (Lu et al., 2014; Carrino et al., 2019). SIRT1 activation also protects against hyperglycemia-induced renal tubular damage via the deacetylation of FOXO3a and the reduction of oxidative stress *in vivo* and *in vitro* (Wang et al., 2017). Natural compound (isoliquiritigenin and dioscin) treatment reduces kidney injury through the SIRT1-dependent mechanism (Song et al., 2019; Huang X. et al., 2020). Here, we observed that *C. cicadae* treatment upregulated SIRT1 and FOXO3a expression and relieved oxidative stress, which in turn regulated autophagy stress that was induced by hypertensive renal injury.

RES, a SIRT1 activator, increased the relative expression of beclin-1 and LC3II, while it decreased p62 expression compared with the untreated control group (Wang et al., 2018; Josifovska et al., 2020). The evidence also demonstrated that RES postponed the development of diabetes by inhibiting autophagy, which included improving cell apoptosis and cellular oxidative stress (Sha et al., 2021). Similar protective effects of RES, namely, reduction of autophagy and the restoration of SIRT1 and FOXO3a levels, were observed in the COPD animal model (Shi et al., 2012). The difference between these studies might be detected using different source cells or animal models. We determined that *C. cicadae* or RES downregulates p62 and disrupts relative expression of beclin-1 and LC3II, indicating that *C. cicadae* controls hypertension-induced autophagic stress by restoring SIRT1 levels in order to inhibit the development of renal fibrosis.

The SIRT1–FOXO3a axis plays a central role in autophagy. An earlier study reported that knocking down SIRT1 enhances cell viability under oxidative stress conditions, triggers nuclear translocation of FOXO3a, and drives FOXO3a acetylation (Han et al., 2016). Moreover, SIRT1 gene silencing disrupts both gAcrp-induced FOXO3a nuclear translocation and LC3II expression (Pun et al., 2015). Our observations are concordant with these viewpoints. For further assessment of the role of FOXO3a, immunofluorescence analysis revealed that the nuclear FOXO3a levels significantly increased compared with cytosolic FOXO3a expression due to its transport from the cytosol to the nucleus following *C. cicadae* treatment. In addition, AngII degraded SIRT1, which in turn increased nuclear translocation of FOXO3a (Supplementary Figure 1A), implying that acetylation of FOXO3a might be involved in AngII-induced fibrosis, and the effect of *C. cicadae* might be mediated by the SIRT1/FOXO3a/ROS-dependent pathway.

*C. cicadae* has been historically used in China for the treatment of CKD. Different biologically active chemical substances, including ergosterol peroxide (EP), N6-(2-hydroxyethyl) adenosine, cordycepic acid, polysaccharides, and effective nucleosides, have been reported (Li et al., 2018). The active components of *C. cicadae* were illustrated using the GC/MS analysis (Supplementary Figure 2). *C. cicadae* also had few sides effects, low toxicity, and no chemical substances (Supplementary Figure 3). Different from cordyceps (*C. sinensis*), *C. cicadae* belongs to the kidney meridian. It is cold in nature according to the theory of TCM, and it is often used to treat CKD without harming the performance of the kidney. TCM has the advantage of being multi-targeted. Recently, *C. cicadae* has been shown to possess a wide range of pharmacological activities in the kidney, which include renal interstitial fibrosis, anti-apoptosis, anti-inflammatory, antiaging, antioxidative stress, and immunoregulatory effects (Xie et al., 2019; Yang et al., 2020; Zhang X. et al., 2021; Chyau et al., 2021). In our study, *C. cicadae* displayed an advantage for reducing renal fibrosis compared with RES. *C. cicadae* inhibited renal fibrosis progression primarily by decreasing ECM deposition through SIRT1-mediated autophagy. The mechanisms may involve the regulation of FOXO3a and oxidative stress. This study provides novel insights into the correlation between TCM and HN.

## Conclusion

This study demonstrated the effects of *C. cicadae* on the pathogenesis of HN and elucidated the underlying mechanisms that may be involved in the SIRT1/FOXO3a/ROS pathway. We revealed that *C. cicadae* upregulates SIRT1/FOXO3a expression, suppresses oxidative stress, decreases ECM accumulation, and controls autophagic stress, which in turn inhibits renal fibrosis and ameliorates hypertensive renal injury. Results of our study provide useful information for the treatment of hypertensive renal injury using TCM by regulating autophagy via the SIRT1 pathway.

## Data Availability

The original contributions presented in the study are included in the article/Supplementary Material, further inquiries can be directed to the corresponding authors.

## Supplementary Material

Supplementary Figure 1
**(A)** Immunofluorescence images show the expression of FOXO3a (green channel) for various times and the location of unclear and cytosolic FOXO3a following AngII treatment (bar = 20 μm).

Supplementary Figure 2 Quality control of *C. cicadae* was evaluated by HPLC GC/MS analysis.

Supplementary Figure 3 LC-MS/MS identification of no chemical substances added into *C. cicadae*.
