# Supplementary figures and images for: Cordyceps cicadae Ameliorates Renal Hypertensive Injury and Fibrosis Through the Regulation of SIRT1-Mediated Autophagy
Source: Front Pharmacol. 2022 Feb 10;12:801094. doi: 10.3389/fphar.2021.801094 (PMC8866973; doi:10.3389/fphar.2021.801094)

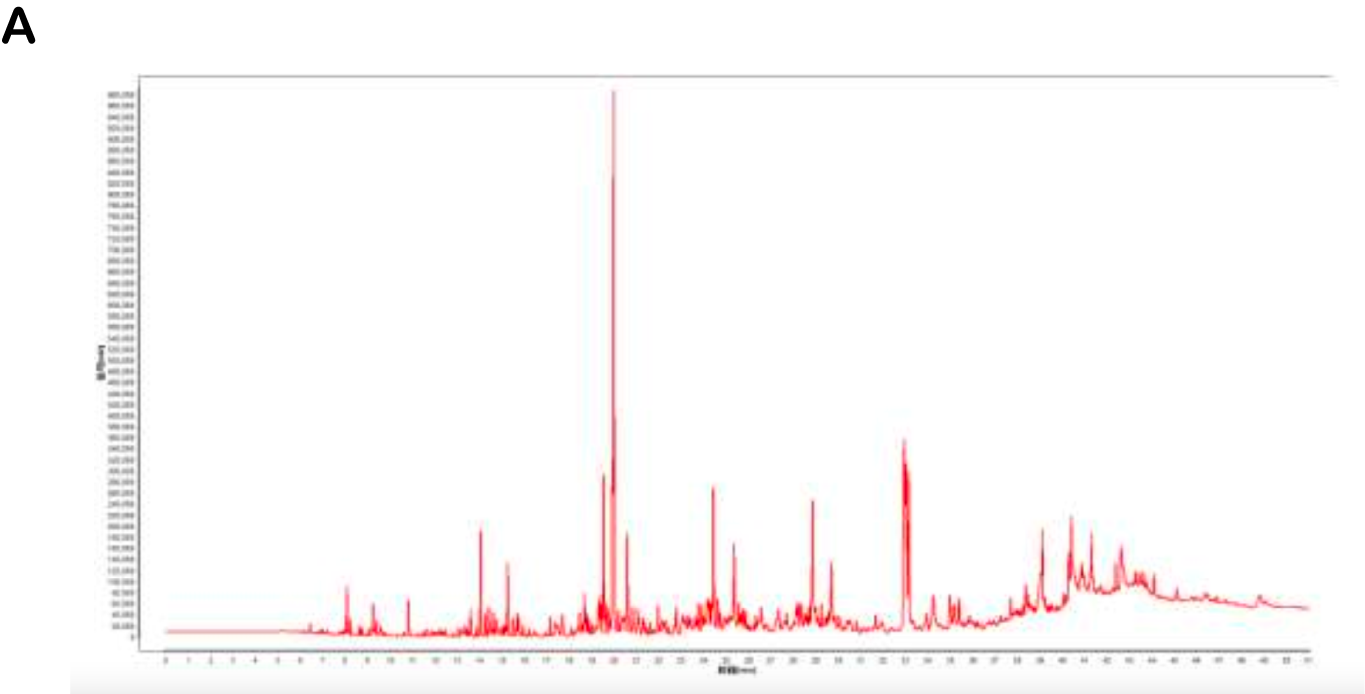

Supplement: Supplementary file 2 [file Image2.TIF]

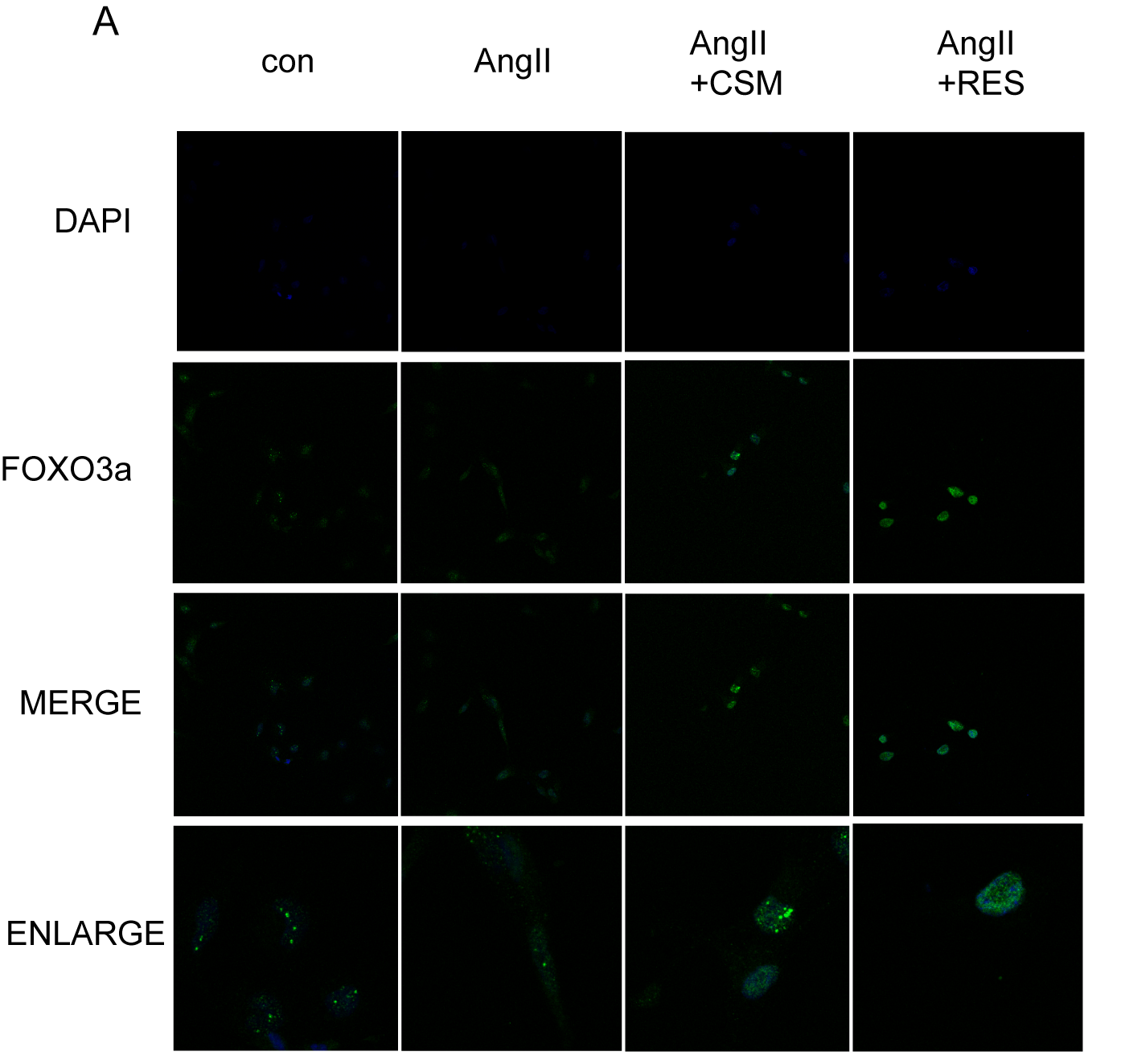

Supplement: Supplementary file 3 [file Image1.TIF]
