# Supplementary material for: Cordyceps cicadae Ameliorates Renal Hypertensive Injury and Fibrosis Through the Regulation of SIRT1-Mediated Autophagy
Source: Front Pharmacol. 2022 Feb 10;12:801094. doi: 10.3389/fphar.2021.801094 (PMC8866973; doi:10.3389/fphar.2021.801094)
Supplement: Supplementary file 4 [file Image3.pdf]

遠傳電信|CMCC
遠傳電信|CMCC ... 169B/s 22:44

TÜVRheinland®
Precisely Right.

杜夫萊茵股份有限公司高雄實驗室
TÜV Rheinland AIMEX Ltd. Kaohsiung Lab

檢測報告
TEST REPORT

報告編號 *Report Number*：15-1202-037-03
送樣日期 *Date Received*：2015/12/02
申請單位 *Applicant*：杜夫萊茵股份有限公司
樣品名稱 *Sample Name*：蟬花虫草纯粉
聯絡電話 *Tel*：07-8317000 ext.100
產品資訊 *Sample Info*：生產日期:2015.05.18
傳真電話 *Fax*：07-8313848
報告日期 *Date Issued*：2015/12/14
申請單位地址 *Applicant Address*：806 高雄市前鎮區南七路3號3樓
檢驗日期 *Test Period*：2015/12/02 ~ 2015/12/14

檢測項目 *Item(s)*	結果 *Result(s)*	備註 *Remark(s)*
西藥成分188項 Western medicine (188)	**陰性 (未檢出)**	細目如附件

~~~以下空白~~~

檢測方法：

以高效能液相層析儀(High Performance Liquid Chromatography)分析。

樣品照片：

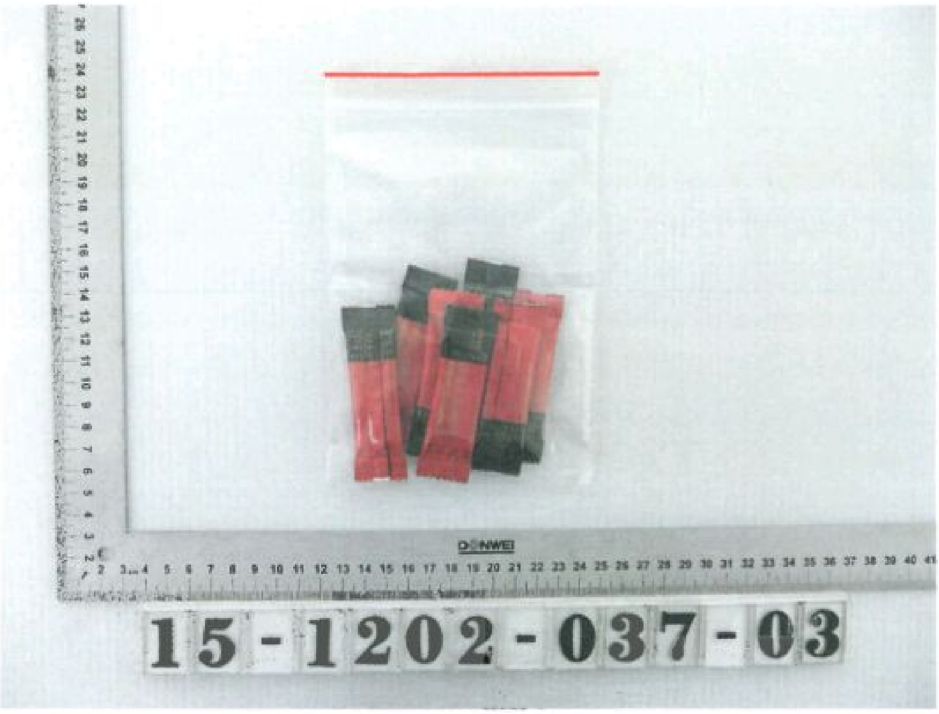

報告簽署人

1.本報告所用樣品與名稱係由委託單位提供，實驗室僅負責檢測分析。
2.檢測結果僅對檢測樣品有效。
3.本報告記載事項僅作為參考資料，不得作為任何商業推銷廣告及訴訟用。
4.本報告內容未經授權不得部份複製，但完整複製除外。
5.本報告經塗改者無效。
6.結果為 N.D.(Non Detected)表未檢出。
7.結果為陽性表檢出，結果為陰性表未檢出。
8.本檢驗未涉及檢體抽樣，報告書僅對該送檢驗檢體負責。
9.檢驗報告僅就委託檢驗者之委託事項提供檢驗結果，至若本產品之合法性，仍應由主管機關依法判斷。
~~~

遠傳電信|CMCC
遠傳電信|CMCC 2.6K/s 22:41

西藥成分188項 Western medicine (188) (附件)

序號	分析項目	M.D.L. (mg/g)
	**類固醇：（9）**	
1	Betamethasone （倍他米松）	2
2	Cortisone acetate（醋酸可的松）	1
3	Dexamethasone （地塞米松）	1
4	Hydrocortisone （氢化可的松）	1
5	Hydrocortisone-21-Acetate （氢化可的松-21-醋酸酯）	1
6	Methylprednisolone（甲基强的松龙）	0.5
7	Prednisolone （泼尼松龙）	0.5
8	Prednisone （泼尼松）	0.2
9	Triamcinolone acetonide（曲安奈德）	1
	**降血脂類：（3）**	
10	Benzafibrate （苯扎贝特）	0.06
11	Clofibrate （降固醇酸）	0.06
12	Gemfibrozil （二甲苯氧庚酸）	0.07
	**荷爾蒙類：（14）**	
13	17a-Ethynylestradiol （17α-乙炔基雌二）	0.16
14	3,3,5-Triiodothyronine free acid （3,3,5甲状腺游离酸）	0.45
15	Carbimazole （卡比马唑）	0.5
16	Diethylstilbestrol （己烯雌酚）	1
17	Estradiol benzoate（苯甲酸雌二醇）	1
18	Estriol （雌激素三醇）	0.25
19	Estrone （雌激素酮）	0.25
20	Methyltestosterone （甲睾酮）	3
21	Progesterone （孕酮）	2.5
22	Testosteronepropionate（丙酸睾酮）	4
23	Danazol （达那唑）	7.5
24	Clomiphene Citrate（克罗米酚柠檬酸盐）	2.5
25	Ethynyl-Androstenediol（乙炔雄二醇）	7.5
26	Formestane （福美坦）	15
	**麻藥拮抗劑：（2）**	
27	Naloxone （纳洛酮）	0.25
28	Naltrexone （纳曲酮）	0.25
	**利尿劑：（8）**	
29	Chlorothiazide （氯噻嗪）	0.2
30	Furosemide （呋塞米）	0.25
31	Hydrochlorothiazide（氢氯噻嗪）	0.6
32	Spironolactone （螺内酯）	1
33	Acetazolamide （乙酰唑胺）	10
34	Metolazone （美托拉宗）	1.75
35	Triamterene （氨苯蝶啶）	0.875
36	Indapamide （吲达帕胺）	0.75
	**抗生素：（30）**	
37	Amoxycillin trihydrate （羟氨苄青霉素三水）	1
38	Ampicillin trihydrate （氨苄青霉素三水）	0.8
39	Chloramphenicol （氯霉素）	0.3
40	Chlortetracycline （金霉素）	0.8
41	Erythromycin estolate（无味红霉素）	3.2
42	Fluconazole （氟康唑）	0.4
43	Griseofulvin （灰黄霉素）	0.3
44	Isoniazid （异烟肼）	0.25
45	Metronidazole （甲硝唑）	1
46	Nalidixic acid （萘啶酸）	1
47	Nitrofurantoin （呋喃妥英）	1
48	Nitrofurazone （呋喃西林）	1
49	Norfloxacin （诺氟沙星）	1
50	Oxolinic acid （恶喹酸）	1
51	Phenazopyridine （非那吡啶）	1
52	Rifampicin （利福平）	0.8

遠傳電信|CMCC
遠傳電信|CMCC
0K/s 22:42

西藥成分188項 Western medicine (188) (附件)

序號	分析項目	M.D.L. (mg/g)
53	Sulfadiazine （磺胺嘧啶）	1
54	Sulfadimethoxine（磺胺二甲氧嘧啶）	0.3
55	Sulfaguanidine （磺胺脒）	0.5
56	Sulfamerazine （磺胺甲基嘧啶）	0.4
57	Sulfamethazine（磺胺二甲嘧啶）	0.45
58	Sulfamethizole （磺胺甲二唑）	0.3
59	Sulfamethoxazole （磺胺甲恶唑）	0.2
60	Sulfamethoxypyridazine（磺胺甲氧哒嗪）	0.4
61	Sulfanilamide （磺胺）	0.4
62	Sulfathiazole （磺胺噻唑）	0.5
63	Sulfisomidine（磺胺二甲氧嘧啶）	1
64	Sulfisoxazole （磺胺异恶唑）	1
65	Tetracycline HCl （盐酸四环素）	0.8
66	Trimethoprim （甲氧苄氨嘧啶）	0.2
	**糖尿病用藥：（7）**	
67	Acetohexamide （醋磺环己脲）	0.5
68	Chlorpropamide （氯磺丙脲）	0.25
69	Glybenclamide （格列本脲）	0.25
70	Metformin （二甲双胍）	0.5
71	Phenformin hydrochloride（盐酸苯乙双胍）	0.5
72	Tolazamide （甲磺氮草脲）	0.13
73	Tolbutamide （甲苯磺丁脲）	0.25
	**抗組織胺類：（8）**	
74	Brompheniramine maleate（马来酸溴苯那敏）	0.13
75	Carbinoxamine maleate（马来酸氯苯吡醇胺）	0.13
76	Chlorpheniramine maleate（马来酸氯苯吡胺）	0.5
77	Cinnarizine （桂利嗪）	0.13
78	Diphenhydramine （苯海拉明）	0.53
79	Diphenidol （二苯哌啶丁醇）	0.14
80	Ketotifen Fumarate（富马酸酮替芬）	0.25
81	Triprolidine （曲普利啶）	0.5
	**腸胃類：（11）**	
82	Atropine （阿托品）	0.4
83	Bisacodyl （比沙可啶）	0.4
84	Buscopan (scopolamine n-butylbromide) （解痉灵）	1
85	Camylofine （卡米罗芬）	1
86	Cimetidine （甲腈咪胺）	0.4
87	DL-Homatropine（氢溴酸后马托品）	0.4
88	Ethaverine （乙基罂粟碱）	0.3
89	Flopropione （三羟苯丙酮）	0.3
90	Metoclopramide （甲氧氯普胺）	0.6
91	Oxyphencyclimine （羟苄利明）	1
92	Propantheline bromide（溴丙胺太林）	0.4
	**精神科類：（13）**	
93	Chlormezanone （氯美扎酮）	0.13
94	Chlorpromazine HCl （盐酸氯丙嗪）	0.25
95	Clordiazepoxide HCl （盐酸氯氮卓）	0.25
96	Diazepam （安定）	0.25
97	Diphenylhydantoin （苯妥英）	0.13
98	Flunitrazepam（FM2）（氟硝西泮）	0.16
99	Fluoxetine （氟西汀）	0.5
100	Meprobamate （氨甲丙二酯）	10
101	Oxazolam （恶唑）	2
102	Phenobarbital （苯巴比妥）	0.25
103	Primidone （扑米酮）	0.25
104	Sulpiride （舒必利）	1
105	Thioridazine （硫利达嗪）	0.5

遠傳電信|CMCC
遠傳電信|CMCC 0K/s 22:42

西藥成分188項 Western medicine (188) (附件)

序號	分析項目	M.D.L. (mg/g)	序號	分析項目	M.D.L. (mg/g)
解熱止痛劑：（26）			133	Diltiazem （地尔硫卓）	0.25
106	Acemetacin （阿西美辛）	0.2			
107	Acetaminophen （对乙酰氨基酚）	0.25	134	Dipyridamole （双嘧达莫）	1
108	Acetylsalicylic acid（乙酰水杨酸）	1	135	Hydralazine HCl （盐酸肼苯哒嗪）	2
109	Aminopyrine （氨基比林）	0.7	136	Methyldopa （甲基多巴）	0.25
110	Benzydamine （苄达明）	0.4	137	Minoxidil （米诺地尔）	0.25
111	Bucetin （布西丁）	0.25	138	Nifedipine （硝苯地平）	0.5
112	Bufexamac （丁苯羟酸）	0.2	139	Pentoxifylline （己酮可可碱）	0.25
113	Diclophenac sodium（双氯芬酸钠）	0.1	140	Prazosin （哌唑嗪）	1
114	Dipyrone （安乃近）	2	141	Propranolol （普萘洛尔）	1
115	Ethoxybenzamide（乙氧基苯甲酰）	0.1	142	Reserpine（蛇根鹼） （蛇根碱）	1
116	Fenbufen （芬布芬）	0.25	神經興奮劑：（7）		
117	Flufenamic acid （氟芬那酸）	0.4	143	Caffeine（咖啡因） （咖啡因）	0.25
118	Flurbiprofen （氟比洛芬）	0.2	144	Ephedrine（麻黄素） （麻黄素）	2.5
119	Ibuprofen （布洛芬）	0.2	145	Methylephedrine （甲基麻黄碱）	1
120	Indomethacin （吲哚美辛）	0.3	146	Phenylephrine （福林）	2
121	Ketoprofen （酮洛芬）	0.18	147	Phenylpropanolamine （苯丙醇胺）	4
122	Mefenamic acid （甲芬那酸）	0.25	148	Theobromine（可可鹼） （可可碱）	1
123	Methylsalicylate （水杨酸甲酯）	0.04	149	Theophylline（茶鹼） （茶碱）	0.13
124	Naproxen （萘普生）	0.4	肌肉鬆弛劑：（5）		
125	Niflumic acid （尼氟灭酸）	0.35	150	Carisoprodol （卡立普多）	10
126	Oxyphenbutazone （羟基保泰松）	0.6	151	Chorzoxazone （氯唑沙宗）	0.63
127	Phenacetin （非那西丁）	0.2	152	Flavoxate （黄酮哌酯）	0.25
128	Phenylbutazone （保泰松）	0.5	153	Mephenesin （甲苯丙醇）	0.13
129	Piroxicam （吡罗昔康）	0.4	154	Orphenadrine citra（枸橼酸苯海拉明）	0.25
130	Salicylamide （水杨酰胺）	0.1	支氣管擴張劑：（4）		
131	Sulindac （舒林酸）	0.2	155	Aminophylline （氨茶碱）	3.38
心臟血管類：（11）			156	Dihydroxypropyltheophylline （喘定二羟丙茶碱喘定） (Diprophylline、Dyphylline）	0.25
132	Atenolol （阿替洛尔）	1	157	Fenoterol （非诺特罗）	4.25

西藥成分188項 Western medicine (188) (附件)

序號	分析項目	M.D.L. (mg/g)	序號	分析項目	M.D.L. (mg/ g)
158	Salbutamol （沙丁胺醇）	1	173	Benzbromarone （苯溴马隆）	0.25
**鎮咳、祛痰劑：（7）**			174	Colchicine （秋水仙碱）	0.25
159	Becantex （双丁萘磺钠）	8	175	Probenecid （丙磺舒）	0.25
160	Bromhexine （溴己新）	2	176	Sulfinpyrazone （苯磺唑酮）	0.25
161	Cloperastine （氯哌斯汀）	0.5	**成癮性麻醉劑、禁用藥物：（6）**		
162	Dextromethorphan （右美沙芬）	1	177	Amphetamine（安非他命）	1
163	Guaifenesin （愈创甘油醚）	0.13	178	Fenfluramine （芬氟拉明）	1
164	Noscapine HCl （那可丁盐酸盐）	1	179	Heroin（海洛因）	1
165	Papaverine（罌粟鹼）（罂粟碱）	0.5	180	Ketamine（K他命）	1
**局部麻醉類：（6）**			181	Methamphetamine（甲基安非他命）	1
166	Benzocaine （苯佐卡因）	0.13	182	Phentermine （芬特明）	0.25
167	Dibucaine （辛可卡因）	0.59	**其他：（6）**		
168	Lidocaine （利多卡因）	0.25	183	Chlorhexidine （洗必泰）	0.4
169	Oxethazaine （奥昔卡因）	0.25	184	Methimazole（抗甲狀腺藥物）	1.3
170	Procaine （普鲁卡因）	5.81	185	Mixed alkyltrimethylammonium bromide （混合烷基三甲基溴化物）	0.8
171	Tetracaine （丁卡）	0.5	186	Nicotine（尼古丁）	0.4
**痛風治療類：（5）**			187	Podophyllotoxin（鬼臼毒素）	0.2
172	Allopurinol （别嘌呤醇）	1	188	santonin （山道年）-驅蟲劑	2.3
